# Legal medicine aspects of female sterilization: our experience

**DOI:** 10.3389/fmed.2023.1198668

**Published:** 2023-07-11

**Authors:** Piergiorgio Fedeli, Stefano Cecchi, Roberto Scendoni, Nunzia Cannovo

**Affiliations:** ^1^University of Camerino, School of Law, Camerino, Italy; ^2^U.O.C. Ginecologia ed Ostetricia, Ospedale Generale Provinciale, AST Macerata, Macerata, Italy; ^3^Department of Law, Institute of Legal Medicine, University of Macerata, Macerata, Italy; ^4^U.O.C. Medicina Legale, AST Macerata, Camerino, Italy

**Keywords:** female sterilization, tubal ligation, hysteroscopic tubal occlusion, non contraceptive benefits, civil proceedings

## Abstract

**Introduction:**

The most frequent sterilization procedures include postpartum tubal ligation, laparoscopic tubal disruption or salpingectomy, and hysteroscopic tubal occlusion. It may be performed via laparoscopy, mini-laparotomy, or hysteroscopy. Safety, efficacy, short-term complications, long-term complications, and non-contraceptive benefits of sterilization are different for each procedure. Female sterilization has become an important professional liability problem in obstetrics and gynecology.

**Materials and methods:**

We analyzed 6 cases of surgical sterilization that have been the subject of civil proceedings. We review indications, contraindications, and complications associated with each sterilization procedure.

**Results:**

In our small number of cases, women who have undergone sterilization performed negligently are entitled to recover damages for wrongful conception, negligence, and wrongful birth. We also consider the issue of female sterilization of minors.

**Discussion:**

Tubal sterilization can be performed with different techniques, chosen in light of the various situations involved, with the goal of reducing as many as possible any failures. Thorough and complete communication of information is of primary importance.

**Conclusion:**

Sterilization is the most widely used birth control method around the world. The procedure is generally safe and highly effective. As reported in the literature, the decision concerning method depends on the setting, the surgeon’s experience, the country’s economic development, and the woman’s preference, but we think that some techniques present a greater risk of failure and expose the surgeon to malpractice litigation.

## 1. Introduction

Female sterilization is performed at the request of women who desire an effective and irreversible form of birth control (1).

The literature describes different techniques (2), characterized by specific profiles of safety and efficacy.

The laparoscopic way is the most common access to perform tubal ligation. In general anestesia, the surgeon can get the better view to the pelvis, and by this technique the woman can come back in few days to her lifestyle. Extimated risk for complications is from 0.9 to 1.6 per 100 procedures (complications from general anestesia, damage to surrounding organs, conversion to open laparotomy, infection, fever), and it increases in case of previous abdominal surgery, obesity (2). There are three options for laparoscopic tubal ligation: electrocoagulation (by monopolar or bipolar energy device), mechianical devices (silicone rubber band, spring-loaded clip or titanium clip; high effectiveness in normal tubes), tubal excision (operator removes part or all the tubes; better choice in abnormal tubes).

The hysteroscopic way for sterilization is to prefer for women who have contrahindications for laparoscopic or laparotomic accesses (3). It is made by introducing substance into the Fallopian tube via tubal ostia, the effect is the block of the tubes.

The laparotomic way is used for sterilization during cesarean section or in women who have controhindicated other way to access the tubes. The risk for complications is 0.39 cases for 100 procedures (fever, blood loss, organ damages) (4). Techniques are: Pomeroy (Mid-isthmic portion of the Fallopian tube is elevated and then folded at the midpoint. One or two rapidly absorbable sutures are tied around the double thickness of the tube, and the folded portion excised sharply), Parkland (an opening is made in an avascular section of the mesosalpinx. Two absorbable suture ties are passed through the opening and used to ligate the proximal and distal ends of the Fallopian tube. The segment (at least 2 cm) between the ties gets excised), Uchida (the midportion of the Fallopian tube is ligated and excised. Then the utero-tubal serosa is hydrodissected, and the proximal tubal stump gets pulled into the mesosalpinx. The peritoneum is closed over the proximal cut end of the tube), Irving (the midportion of the Fallopian tube is ligated and excised. Then the proximal tubal stump is inserted into an incision made into the myometrium and securely sutured to bury the proximal stump in the myometrium), Distal Fimbriectomy (the fimbriated end of the Fallopian tube is ligated and excised), Complete Salpingectomy (The entire Fallopian tube to the (except the interstitial portion) is excised; this can be done with suture ligation, laparoscopic bipolar devices).

Reported 10-years pregnancy risk by technique (2, 4): salpingectomy 7.5/1000 procedures, bipolar coagulation 24.8/1000, silicone band 17.7/1000, spring clip 36.5/1000.

Reported ectopic pregnancy risk by techinique (2, 5, 6): salpingectomy 1.5/1000 procedures, bipolar coagulation 17.1/1000, silicone band 7.3/1000, spring clip 8.5/1000.

Since 2000, transcervical tubal sterilization has been approved in Australia, Singapore, Europe, Canada, and the United States (7).

Laws and regulations surrounding tubal sterilization can vary between European countries (8). In Poland and Lithuania, for example, female sterilization is very restrictive; it may only be performed to preserve a woman’s life or health. However, some aspects that may be considered in relation to singular law include:

a. age limit: in Europe the age of majority is required (in Portugal the minimum age limit is 25 years old);
b. informed Consent: it is generally required in all European countries for any medical procedure, including tubal sterilisation;
c. waiting period: some countries may provide mandatory or recommended waiting periods (France, Germany, Italy) between the moment of informed consent and the actual procedure; this allows people to carefully consider their decision and prevent hasty decisions;
d. spousal consent: generally not required;
e. counseling: some countries may require individuals to receive counseling or psychological evaluation before tubal sterilization; this is to ensure that people have considered all available options and fully understand the irreversible nature of the procedure.

It should also be noted that the increased efficacy and acceptability of long-acting reversible contraceptive methods (LARCs) has contributed in a trend towards declining sterilisation rates in some regions, e.g., the United Kingdom, in favour of LARCs (9).

## 2. Materials and methods

We analyzed 6 cases of surgical sterilization that have been the subject of civil proceedings. We review indications, contraindications, and complications associated with each sterilization procedure (Supplementary Table S1 Case analyses).

## 3. Results

In the first clinical case, the technical consultant (TC) of the Judge reported elements of medical malpractice due to imprudence and negligence, given that in that circumstance, the physician had chosen the Pomeroy technique, known to be marked by failure (albeit a low rate), rather than the Irving or Uchida methods, which would have offered greater guarantees of success. In addition, during the second procedure performed on the occasion of the cesarean section, physicians observed imperfect execution of the surgical sterilization as the cause of the subsequent pregnancy.

In addition, the patient had not been properly informed of the percentage of failures of the technique chosen.

In the second clinical case, the TC of the Judge confirmed as appropriate the choice not to limit the procedure of tubal sterilization to mere tubal excision, but to perform obliteration of the stumps with diathermocoagulation, in order to obtain the best results.

On the basis of the description of the operation, it appeared to have been conducted according to the procedure of the method chosen; the failure could be due to incorrect performance of the operation or to other causes, such as recanalization due to phlogosis or other reasons.

After tubal sterilization, recanalization and thus intrauterine or extrauterine pregnancy is very rare but not impossible.

In the third clinical case, it may be hypothesized that the tubal recanalization (fistulization) was not due to incorrect performance of the technique. Instead, it appears that the physician informed the patient that one technique would be used, but then performed a different one, which had a higher probability of failure.

The operation in the fourth case was not well described, but it seems that the physician simply applied thread (it is not specified whether it was absorbable or non-absorbable) around the tubes and then tied them. No other measures to minimize failure were taken, even though the woman’s obesity and her previous operations and probable adhesions dictated the need to do so.

In the fifth case, the surgery records indicated in generic terms that the tubes were tied, but during the second operation, they were found to be open, and the physicians then performed bilateral salpingectomy. Apparently, the recanalization was due to an error in carrying out the first procedure.

In the sixth case, a technique marked by failures was performed initially, and then corrected in a second operation with one with more positive outcomes.

In all six cases, the techniques chosen by the surgeons were appropriate for the situations, which presented no contraindications. However, among the laparotomic techniques, the only one that offers significant advantages in terms of results is bilateral salpingectomy, which in fact was chosen to correct the situation in the fifth case.

## 4. Discussion

So-called “reversible” contraceptive methods are assigned a failure percentage, the Pearl Index (10), indicating the number of undesired pregnancies/100 women/year of use, with measurements for perfect use and for typical use, which differ greatly, according to the woman’s ability to follow to perfection the instructions for the method.

So-called “irreversible” contraceptive methods (such as the inaccurately termed “tubal sterilization”) are also assigned a failure percentage, though the woman’s compliance is not a factor, and thus the measurement considers perfect use of the technique. Though tubal sterilization can be defined as very much effective, the risk of failure is higher than generally reported (11). It must be stated clearly that a failure rate of 0 exists for no contraceptive methods except absolute abstinence, or occurs only in the absence of normal anatomy or function of the genital apparatus.

As indicated above, there are surgical female sterilization techniques (Pomeroy and Parkland) and those that involve access to the genital apparatus to place clips or rings along the tube, or to apply devices, such as an IUD, to the lumen of the tube.

In the six cases examined, the Pomeroy technique (12), which is best known among physicians, was the most commonly used. In fact, the Pomeroy and Parkland techniques are the most often chosen, given that they are equally simple and effective and have the same frequency of complications (13).

There are contraindications to these procedures, for example, pathological obesity or extensive adhesions due to a series of operations. In addition, a good outcome can be compromised by salpingitis, peritonitis and phlogosis because they can cause necrotic processes at the site of tubal sterilization (14).

Given the relative reversibility of the Pomeroy and Parkland techniques, they could be an option for minors when it is indispensable to proceed with a form of sterilization to safeguard the health of the young woman (15–17).

Malpractice litigation involving adult women is commonly related to the failure percentage of the method, poor surgical technique, or inadequate information and consent (18).

Surgeons should be scrupulous in compiling their reports, describing in detail the various stages of the technique performed, rather than just jotting down fragments of the operation process or providing an epicrisis such as “surgery according to Pomeroy or Parkland method.” It is in the surgeon’s best interests to document in detail in the patient record every phase of the procedure, given the juridical importance of this record (19) for the physician’s defense during malpractice lawsuits, as it could serve to demonstrate the effective execution and completeness of the technique performed. It is more likely that a pregnancy post-Pomeroy sterilization would be due to a spontaneous process of tubal recanalization (not frequent, but possible) rather than to the surgeon’s incomplete or technically incorrect performance of the procedure.

Therefore, the informative phase of the woman is of fundamental importance.

It is well known that the phase of information and that of acquiring consent do not coincide but are the first preparatory to the other.

However, communication is not just about giving information; it also involves listening to the patient, answering her questions and verifying that she understood what she was told.

Information to the patient is itself a health service, as required by law (20). Physicians have a professional duty to explain and initiate a discussion on the risks, benefits and possible alternatives for all surgical/pharmaceutical procedures that may be proposed to the patient. In fact, patients may not be able to assess the risks and should be guided in their choices in their best interests (21).

Only at the end of this clear and exhaustive communication process will the patient be able to express a free and informed consent (22).

As reported in the literature (23, 24), exhaustive information and a good communication relationship between physician and patient seem to influence the likelihood of malpractice complaints.

In our small number of cases, women who underwent sterilization performed negligently are entitled to recover damages for wrongful conception, negligence and wrongful birth.

In common law countries, as in many civil law ones, the term “wrongful birth” (25, 26) refers to an involuntary birth that occurs as a result of medical malpractice or a not a success of some form of birth control procedure (27). Italian legislation does not raise the problem of unwanted birth as a reason for compensation. Italian judges have favored an analysis that placed the accent not so much on the “birth” of the child in and of itself and on the economic prejudice deriving from the maintenance of the child, but rather on the denial of the possibility of exercising the right to pregnancy termination which the law attributes to the pregnant woman as the right to choose between the continuation of the life of the conceived and the protection of health (28). In this way, wrongful birth claims can also be made when the child is born healthy.

Given that failures are statistically more frequent when the procedure is performed at the end of a cesarean section or in any case a birth, it is usually suggested to allow a few days to pass before proceeding with the tubal sterilization (11). For this same reason, a Cesarean section should not be performed exclusively for the reason of sterilization, considering also that it would increase the risks inherent in surgery, without proper motivation.

Tubal sterilization is generally considered for women at least 30 years old who have at least two children, and who are profoundly convinced that they want this done, in full agreement with their partner. In fact, negative psychological effects have been reported post-sterilization when the women realized they had made an inappropriate decision, perhaps related to particular circumstances (29, 30).

In conclusion, tubal sterilization can be performed with different techniques which can be modified, also in light of the various situations involved, with the goal of reducing the risk of failure as much as possible (31–33). Thus, as indicated above, this procedure to exclude a pregnancy may, albeit not frequently, fail independently of the technique adopted. Thorough and complete communication of information is of primary importance. The woman must be informed of the fact that the closure of the Fallopian tubes does not definitively and with absolute certainty exclude the possibility of conception after intercourse in absence of other birth control methods.

## 5. Conclusion

Sterilization has remained the most usually used method around the world (34). The procedure is generally safe and highly effective. As reported in the literature, the decision concerning method depends on different aspects, but we think that some techniques present a greater risk of failure and expose the surgeon to a claim for damages.

The female sterilization procedure requires shared decision-making between the patient and her healthcare provider. The informed consent process for sterilization is primary for avoiding professional liability charges.

## Data availability statement

The original contributions presented in the study are included in the article/Supplementary material, further inquiries can be directed to the corresponding author.

## Author contributions

PF found clinical cases. SC wrote the introduction section. RS reworked the discussion section. NC wrote the first version of the article. All authors contributed to the article and approved the submitted version.

## Conflict of interest

The authors declare that the research was conducted in the absence of any commercial or financial relationships that could be construed as a potential conflict of interest.

## Publisher’s note

All claims expressed in this article are solely those of the authors and do not necessarily represent those of their affiliated organizations, or those of the publisher, the editors and the reviewers. Any product that may be evaluated in this article, or claim that may be made by its manufacturer, is not guaranteed or endorsed by the publisher.

## References

[1] Mieke C Eeckhaut W Sweeney MM. The perplexing links between contraceptive sterilization and (dis)advantage in ten low-fertility countries. Popul Stud (Camb). (2016) 70:39–58. doi: 10.1080/00324728.2015.1122209, PMID: 26792541 PMC4798874

[2] American College of Obstetricians and Gynecologists' Committee on Practice Bulletins—Gynecology. ACOG practice bulletin no. 208: benefits and risks of sterilization. Obstet Gynecol. (2019) 133:e194–207. doi: 10.1097/AOG.0000000000003111, PMID: 30640233

[3] Bouillon K Bertrand M Bader G Lucot JP Dray-Spira R Zureik M. Association of Hysteroscopic vs laparoscopic sterilization with procedural, gynecological, and medical outcomes. JAMA. (2018) 319:375–87. doi: 10.1001/jama.2017.21269, PMID: 29362796 PMC5833563

[4] Huber AW Mueller MD Ghezzi F Cromi A Dreher E Raio L. Tubal sterilization: complications of laparoscopy and minilaparotomy. Eur J Obstet Gynecol Reprod Biol. (2007) 134:105–9. doi: 10.1016/j.ejogrb.2006.06.016, PMID: 16872736

[5] Peterson HB Xia Z Wilcox LS Tylor LR Trussell J. Pregnancy after tubal sterilization with bipolar electrocoagulation. U.S. collaborative review of sterilization working group. Obstet Gynecol. (1999) 94:163–7. doi: 10.1016/s0029-7844(99)00316-6, PMID: 10432120

[6] Satoh K Osada H. Post-tubal ligation syndrome. Ryoikibetsu Shokogun Shirizu. (1993) 1:772–3. 7757737

[7] Cullins V. Global Library women's Medicine (2008).

[8] Scott A Glasier A. Contraceptive sterilization: global issues and trends. Bull World Health Organ. (2003) 81:146.

[9] Lawrie TA Kulier R Nardin JM. Techniques for the interruption of tubal patency for female sterilisation. Cochrane Database Syst Rev. (2016) 2016:CD003034. doi: 10.1002/14651858.CD003034.pub4, PMID: 27494193 PMC7004248

[10] Shoupe D. The handbook of contraception: Evidence based practice recommendations and rationales. Totowa, New Jersey: Springer International Publishing (2020).

[11] Peterson HB Xia Z Wilcox LS. The risk of pregnancy after tubal sterilization: findings from the U.S. collaborative review of sterilization. Am J Obstet Gynecol. (1996) 174:1161–70. doi: 10.1016/S0002-9378(96)70658-0, PMID: 8623843

[12] Ferraris G. Chirurgia ginecologica ed ostetrica In: . Trattato di Tecnica Chirurgica Vol. 2. Torino: UTET (1986).

[13] Bartz D Greenberg JA. Sterilization in the United States. Rev Obstet Gynecol. (2008) 1:23–32. PMID: 18701927 PMC2492586

[14] Dessole S. Capobianco G. (2006 – 2009). “Tecniche laparotomiche per patologia benigna: la sterilizzazione tubarica” In Ginecologia e ostetricia, Vol. II. Roma: Verduci Editore.

[15] Godin PA Konstantinos S Rege G Demir S Charitidou E Wery O. Laparoscopic reversal of tubal sterilization; a retrospective study over 135 cases. Front Surg. (2018) 5:79. doi: 10.3389/fsurg.2018.00079, PMID: 30687715 PMC6333701

[16] Jayakrishnan K Baheti SN. Laparoscopic tubal sterilization reversal and fertility outcomes. J Hum Reprod Sci. (2011) 4:125–9. doi: 10.4103/0974-1208.92286 22346079 PMC3276946

[17] Karayalcin R Ozcan S Tokmak A Gürlek B Yenicesu O Timur H. Pregnancy outcome of laparoscopic tubal reanastomosis: retrospective results from a single clinical Centre. J Int Med Res. (2017) 45:1245–52. doi: 10.1177/0300060517709815, PMID: 28534697 PMC5536424

[18] Fedeli P Guida M Giugliano P Mazzarelli LL D’Apuzzo A Scendoni R. Femoral nerve injury in gynecologic surgery: medico-legal issues for best surgical practices. Gynecol Surg. (2021) 18:6. doi: 10.1186/s10397-021-01086-7

[19] Occorsio V. Cartella clinica e responsabilità medica. Milano: Giuffrè Editore (2011).

[20] Law 22 December 2017, n. 219 (2017). Norme in materia di consenso informato e di disposizioni anticipate di trattamento. Official Gazzette, n.12 of the 16 January 2018. Available at: https://www.gazzettaufficiale.it/eli/id/2018/1/16/18G00006/sg

[21] Vergallo GM Zaami S. Guidelines and best practices: remarks on the Gelli-Bianco law. Clin Ter. (2018) 169:e82–5. doi: 10.7417/T.2018.2059, PMID: 29595871

[22] Gullo G Scaglione M Buzzaccarini G Laganà AS Basile G Chiantera V. Cell-free fetal DNA and non-invasive prenatal diagnosis of Chromosomopathies and pediatric monogenic diseases: a critical appraisal and medicolegal remarks. J Pers Med. (2023) 13:1. doi: 10.3390/jpm13010001, PMID: 36675662 PMC9862851

[23] Hamasaki T Takehara T Hagihara A. Physicians' communication skills with patients and legal liability in decided medical malpractice litigation cases in Japan. BMC Fam Pract. (2008) 9:43. doi: 10.1186/1471-2296-9-43, PMID: 18652700 PMC2515845

[24] Hamasaki T Hagihara A. Physicians' explanatory behaviours and legal liability in decided medical malpractice litigation cases in Japan. BMC Med Ethics. (2011) 2011:7. doi: 10.1186/1472-6939-12-7 PMC3112190 21510891

[25] Bopp J Bostrom BA McKinney DA. The rights and wrongs of wrongful birth and wrongful life: a jurisprudential analysis of birth related torts. Duq L Rev. (1988) 27:461.

[26] Hassan M Chitty L Reardon H. Wrongful birth: clinical settings and legal implications. Semin Fetal Neonatal Med. (2014) 19:312–6. doi: 10.1016/j.siny.2014.08.006, PMID: 25186057

[27] Frati P Fineschi V di Sanzo M la Russa R Scopetti M Severi FM. Preimplantation and prenatal diagnosis, wrongful birth and wrongful life: a global view of bioethical and legal controversies. Hum Reprod Update. (2017) 23:338–57. doi: 10.1093/humupd/dmx002, PMID: 28180264

[28] Cassazione civile, sez. III, sentenza 24/10/2013 n° 24109. Available at: https://www.quotidianosanita.it/allegati/allegato46876.pdf

[29] Bottiglioni F Guerresi E. La sterilizzazione maschile e femminile. Cosenza: Editoriale Bios (1986).

[30] Hayford SR Kissling A Guzzo KB. Changing educational differentials in female sterilization. Perspect Sex Reprod Health. (2020) 52:117–27. doi: 10.1363/psrh.12137, PMID: 32462730 PMC7669611

[31] Zerden ML Castellano T Doll KM Stuart GS Munoz MC Boggess KA. Risk-reducing salpingectomy versus standard tubal sterilization: lessons from offering women options for interval sterilization. South Med J. (2019) 111:173–7. doi: 10.14423/smj.0000000000000779 PMC6419963 29505655

[32] Tasset J Jensen JF. Efficacy of tubal surgery for permanent contraception: considerations for the clinician. Open Access J Contracept. (2023) 14:53–9. doi: 10.2147/OAJC.S385255 36959873 PMC10029365

[33] McMartin K. Hysteroscopic tubal sterilization: an evidence-based analysis. Ont Health Technol Assess Ser. (2013) 13:1–35. PMID: 24228084 PMC3819111

[34] Chapman L Magos A. Currently available devices for female sterilization. Expert Rev Med Devices. (2014) 2:623–34. doi: 10.1586/17434440.2.5.623 16293074

