# Supplementary material for: Legal medicine aspects of female sterilization: our experience
Source: Front Med (Lausanne). 2023 Jul 11;10:1198668. doi: 10.3389/fmed.2023.1198668 (PMC10367092; doi:10.3389/fmed.2023.1198668)
Supplement: Supplementary file 1 [file Table_1.DOC]

Supplementary Material

# Legal medicine aspects of female sterilization: our experience

Piergiorgio Fedeli^1^ †, Stefano Cecchi^2^ †, Roberto Scendoni^3^ †, Nunzia Cannovo^4*^ †

^1^University of Camerino, School of Law, Camerino, Italy;

^2^U.O.C. Ginecologia ed Ostetricia, Ospedale Generale Provinciale, AST Macerata, Macerata, Italy

^3^ Institute of Legal Medicine, Department of Law, University of Macerata, Macerata, Italy

^4^U.O.C. Medicina Legale, AST Macerata, Camerino, Italy

*** Correspondence:**Nunzia Cannovo
nunzia.cannovo@gmail.com

# Table n. 1 Cases analyses

| CASE | Technique described in the medical record of the 1^st^ procedure | Indication | Contraindication | Complication | Technique described in the medical record for the 2^nd^ procedure |
| --- | --- | --- | --- | --- | --- |
| I | Bilateral tubal sterilization using classic surgical technique | indicated | none | Unexpected pregnancy | The right tube appears to be excised; the left tube instead shows silk thread sutures. The tube is tied and excised using the  Pomeroy technique |
| II | Tubal sterilization by excision using Pomeroy technique and DTC of the stumps | indicated | none | Unexpected pregnancy | Tubal excision and obliteration of the stumps with diathermocoagulation |
| III | Tubal sterilization using Pomeroy technique | indicated | none | Unexpected pregnancy | Observed previous bilateral tubal ligation by Pomeroy technique; probable recanalization of the right tube. Performed bilateral double legation, coagulation and re-excision of the intermediate section of the tube using the Parkland technique. The tubal fragments were removed and sent for histology. |
| IV | Bilateral tubal ligation | indicated | none | Unexpected pregnancy | Medical records provide no information on what was observed.  Performed tubal ligation and excision using the Pomeroy technique. |
| V | Tuballigation | indicated | none | Unexpected pregnancy | Subsequent bilateral salpingectomy procedure |
| VI | Tubal sterilization using the Pomeroy technique | indicated | none | Unexpected pregnancy | Recanalization of the tubes; the patient chose not to pursue sterilization |
